# Disulfiram Exhibits Potent Copper-Dependent Antimicrobial Activity Against *Mycoplasma genitalium*

**DOI:** 10.1093/ofid/ofag042

**Published:** 2026-03-30

**Authors:** Li Xiao, Grayson R Gimblet, Suzanne E Lapi, William M Geisler, James Daubenspeck, T Prescott Atkinson

**Affiliations:** Department of Medicine, University of Alabama at Birmingham, Birmingham, Alabama, USA; Department of Radiology, University of Alabama at Birmingham, Birmingham, Alabama, USA; Department of Radiology, University of Alabama at Birmingham, Birmingham, Alabama, USA; Department of Medicine, University of Alabama at Birmingham, Birmingham, Alabama, USA; Department of Pediatrics, University of Alabama at Birmingham, Birmingham, Alabama, USA; Department of Pediatrics, University of Alabama at Birmingham, Birmingham, Alabama, USA

**Keywords:** copper-64, disulfiram-copper, drug repurposing, drug resistance, *Mycoplasma genitalium*

## Abstract

**Background:**

*Mycoplasma genitalium* (Mgen) is a sexually transmitted urogenital pathogen. Studies in sexual health clinic settings have demonstrated that Mgen infection, with similar prevalence to *Chlamydia trachomatis* infection, is becoming increasingly resistant to the limited classes of antibiotics with in vitro and clinical activity. Disulfiram (DS) is a Food and Drug Administration–approved drug for treatment of alcohol dependence that shows promise for repurposing as an antimicrobial agent.

**Methods:**

Minimum inhibitory concentration was performed via standard methods for human mycoplasmas and ureaplasmas as established by guideline M43-A of the Clinical and Laboratory Standards Institute. Mgen strain G37 was used as a part of a colorimetric checkboard assay to explore the mechanism of growth inhibition with DS, with emphasis on the role of copper on DS antimicrobial activity. Trace concentrations of radioactive ^64^Cu were used to understand Cu^2+^ trafficking and probe the antimicrobial mechanism of DS for strain G37 and 2 DS-resistant mutants (D3 and D4).

**Results:**

DS exhibits in vitro antimicrobial activity against Mgen isolates with low minimum inhibitory concentration values, regardless of susceptibility to other antibiotics. Growth inhibition studies with DS demonstrated that antimicrobial activity is copper dependent. Studies with ^64^Cu showed that DS greatly enhances copper uptake but that impaired DS-mediated copper uptake is not the mechanism for DS-resistance in Mgen mutants D3 and D4.

**Conclusions:**

DS exhibits potent copper-dependent antimicrobial activity against Mgen. DS or a similar compound could be developed as therapy for drug-resistant Mgen infections.

## Teaser

*Mycoplasma genitalium* is a sexually transmitted urogenital pathogen, with similar prevalence to *Chlamydia trachomatis*, that is becoming increasingly resistant to antibiotics. We show that disulfiram exhibits copper-dependent in vitro antimicrobial activity against *M genitalium* isolates regardless of susceptibility to other antibiotics.

*Mycoplasma genitalium* (Mgen) is a highly genome-reduced, cell wall–less human urogenital pathogen that can cause urethritis, cervicitis, and pelvic inflammatory disease [1]. The prevalence of Mgen infection in the general population approaches that of *Chlamydia trachomatis* infection, but unlike *C trachomatis*, Mgen is developing antimicrobial resistance to the limited classes of antibiotics that have been shown to have in vitro and clinical activity against it [1–4]. Increasingly, cases are being reported of chronic infection with strains of Mgen that are resistant to all known therapies; therefore, new therapeutic options for Mgen are urgently needed [5].

**Figure 1.**
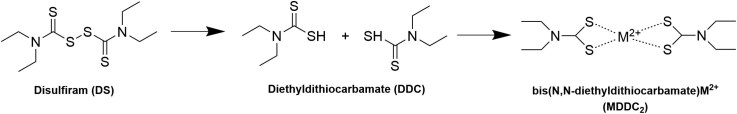
Structure of disulfiram (DS). DS is a Food and Drug Administration–approved drug historically used for the treatment of alcohol use disorder. DS is a prodrug for diethyldithiocarbamate, which acts as a chelator for divalent metal ions (Cu^2+^, Zn^2+^).

Disulfiram (DS; tetraethylthiuram disulfide) is a Food and Drug Administration–approved drug for the treatment of alcoholism. The pharmacotherapy of DS is dependent on the irreversible inhibition of aldehyde dehydrogenase, leading to a toxic buildup of acetaldehyde (Figure 1) [6]. DS is a prodrug that is rapidly reduced in the intestinal tract to 2 molecules of diethyldithiocarbamate (DDC), which, in a complex with metals such as copper and zinc, is highly hydrophobic and rapidly absorbed [7, 8]. The pharmacologic effects of DS are mainly exerted by DDC-metal complexes [7]. Excretion of DS metabolites is largely via the urinary tract [9].

We previously described the effect of copper-chelating drugs, including DS, on several human mollicute species [10]. In the current study, we examined the effects of DS and DDC on the growth of Mgen in vitro and find that they exhibit a low minimum inhibitory concentration (MIC), irrespective of whether Mgen strains were susceptible or resistant to tetracyclines, macrolides, or fluoroquinolones. Furthermore, the inhibitory effect on growth in culture can be reversed with bathocuproine sulfonate (BCS), a membrane-impermeable copper chelator, indicating that the inhibitory effect of DS and DDC is copper dependent. Finally, we demonstrate that DS and DDC induce concentration-dependent uptake of ^64^Cu^2+^ by Mgen, supporting the transport of copper ions across the bacterial membrane as a likely mechanism of the antimicrobial action of these compounds.

## MATERIALS AND METHODS

### Organisms and Reagents

Antibiotic MIC testing was performed for 6 strains of Mgen (G37 and 5 clinical isolates), *Mycoplasma pneumoniae*, *Mycoplasma hominis*, and *Ureaplasma urealyticum*. Strains of Mgen G37 (ATCC 33530), *M pneumoniae* M129 (ATCC 29342), *M hominis* PG21 (ATCC 23114), and *U urealyticum* serovar 9 (ATCC 33175) were from American Type Culture Collection (ATCC). Mgen clinical strains were obtained from the UAB Diagnostic Mycoplasma Laboratory.

*Mycoplasma* species were cultured in SP4 medium and *U. urealyticum* was cultured in 10B medium (both prepared and quality control tested by the UAB Diagnostic Mycoplasma Laboratory) containing 300 µg/L of ampicillin. DS-resistant mutant sublines D3 and D4 were generated by growing the G37 parent strain in gradually increasing concentrations of DS in SP4 medium. DS (tetraethylthiuram disulfide, 86720; Sigma) was dissolved at 5 mg/mL (16.9 mM) in ethanol. DDC (sodium diethyldithiocarbamate trihydrate, 228680; Sigma) was dissolved at 3.8 mg/mL (16.9 mM) in ethanol. BCS (bathocuproinedisulfonic acid disodium salt hydrate, 146625; Sigma) was dissolved at 5.7 mg/mL (10 mM) in Milli-Q water. Cu_2_SO_4_ (Sigma) was dissolved at 204.8 mM in water.

### Antimicrobial Susceptibility Testing (AST)

Inoculum preparation, broth microdilution, MIC assays, and quality control procedures were performed by established methods for human mycoplasmas by guideline M43-A of the Clinical and Laboratory Standards Institute (CLSI) with some modifications necessary to accommodate the slow growth of Mgen as previously reported [11]. Testing was performed in SP4 medium for azithromycin (macrolide class), levofloxacin (fluoroquinolone class), tetracycline (tetracycline class), and DS. Currently there are no CLSI interpretation criteria for Mgen. In this study, the criteria for *M pneumoniae*, the closest species to Mgen, was used for reference [11]. The MIC cutoff values for susceptibility to azithromycin, levofloxacin, and tetracycline are ≤0.5, ≤1, and ≤2 mg/L.

### Growth Inhibition Curves

For measurement of growth inhibition curves, G37 was cultured in 5-mL sterile culture tubes containing SP4 broth until initial color change was detected; then, 10-µL aliquots were used to seed 96-well cell culture plates. In a typical checkerboard assay, the plate was loaded with 100 µL of SP4, and reagents diluted in SP4 were added in 100 µL to the top row and serially diluted vertically, followed by the addition of the second reagent in 100 µL to the first column and serially diluted horizontally as indicated. Finally, each assay well was seeded with 10 µL of G37. Positive control wells contained no reagents, and negative control wells contained no bacteria. Plates were taped and sealed in plastic bags with a moist gauze and incubated at 37 °C until the positive control wells developed color change indicating stationary growth phase. Plates were read in a microplate reader at 450 nm with growth-induced color change (yellow) producing a progressive increase in optical density readings.

### Production of ^64^Cu

Copper-64 (^64^Cu, t_1/2_ = 12.7 hours, β^+^ = 17.9%) was produced as CuCl_2_ through the irradiation of enriched nickel-64 (^64^Ni) [12] by the UAB Cyclotron Facility on an ACSI TR-24 cyclotron (Advanced Cyclotron Systems Inc), as previously described [13]. ^64^Cu was used within 48 hours of production.

### General ^64^Cu Uptake Protocol

For each experiment assessing ^64^Cu uptake, G37 was grown to an exponential phase (SP4 broth color change from red to orange) in 80 to 120 mL of culture, and organisms were scraped and collected by centrifugation at 4800*g* for 20 minutes at 4 °C. A suspension of G37 was spun down at 11 000g for 2 minutes at 24 °C and washed with phosphate-buffered saline (PBS; ×3) prior to beginning the incubation. For each 300-µL reaction, aliquots of 25 µL of resuspended G37 in PBS and 50 µL of ^64^Cu diluted to 0.3 µCi/mL in PBS were added. Stocks of CuSO4, DS, and its metabolite DDC were diluted in PBS and added to the reaction such that addition would not exceed 300 µL. PBS was added to reach a final volume of 300 µL. The reaction was incubated for 1 hour at room temperature in an end-over-end rotator. After incubation, bacteria were spun down at 11 000*g* for 2 minutes at 4 °C. The supernatant was aspirated and resuspended in ice-cold PBS (×3). After the final wash, the supernatant was aspirated; samples were placed on a gamma counter and decay corrected; and percentage associated was measured as a percentage of the total.

### Statistics

Statistical evaluations were performed in Prism 9 (GraphPad Software). Comparisons were made with 2-tailed unpaired *t* tests (α = .05) or ordinary 1-way analysis of variance where applicable.

## RESULTS

### Mollicute MIC Testing

Antibiotic MIC testing was performed by standard methods for human mycoplasmas and ureaplasmas established by CLSI guideline M43-A [11] (Table 1). Testing was performed in SP4 medium for azithromycin (macrolide class), levofloxacin (fluoroquinolone class), tetracycline (tetracycline class), and DS for 6 strains of Mgen (G37 and 5 clinical isolates) and a closely related mycoplasma, *M pneumoniae*, with comparison with 2 more distantly related mollicutes, *M hominis* and *U urealyticum*. Previous analysis in our group showed that SP4 contains about 0.2µM Cu^2+^ [10].

**Table 1.** MIC Testing of *Mycoplasma genitalium* Isolates and Other Mollicutes Against Disulfiram and Other Antimicrobial Classes

			MIC, mg/L			
Organism: Strain	Source	Susceptibility	Azithromycin	Levofloxacin	Tetracycline	DS
*M genitalium*						
33530 (G37)	ATCC	S	≤0.004	1	0.25	0.016
M2321	Clinical	S	≤0.004	1	0.25	0.063
M2341	Clinical	S	≤0.004	0.25	2	0.031
M6282	Clinical	S	≤0.004	0.25	0.125	0.063
UAB-75956	Clinical	MR/FQR/TR	≥32	8	16	0.031
UAB-85276	Clinical	MR, FQR	2	2	2	0.031
*M pneumoniae*						
29342 (M129)	ATCC	S	≤0.001	1	0.25	0.063
*M hominis*						
23114 (PG21)	ATCC	S	2	1	0.25	4
*U urealyticum*						
33175 (Serovar 9)	ATCC	TR	1	1	16	8

Currently there are no Clinical and Laboratory Standards Institute interpretation criteria for *M genitalium*. In this study, the criteria for *M pneumoniae* were used for reference [11]. The cutoff values of susceptibility to azithromycin, levofloxacin, and tetracycline are ≤0.5, ≤1, and ≤2 mg/L.

Abbreviations: DS, disulfiram; FQR, fluoroquinolone resistant; MIC, minimal inhibitory concentration; MR, macrolide resistant; S, susceptible; TR, tetracycline resistant.

We found that DS exhibits antimicrobial effects against all Mgen strains, with MICs ranging from 0.031 to 0.063 mg/L (104–212 nM). The MIC with DS for *M pneumoniae* (M129), a closely related species, was similar to Mgen strains, while that for the 2 more distantly related human urogenital mollicutes *M hominis* and *U urealyticum* was >60-fold higher. Similar findings were obtained in a previous study from this laboratory [10]. Notably, all Mgen strains were DS sensitive despite resistance to other antibiotics in 2 clinical strains, UAB-75956 and UAB-85276.

### Mgen Growth Inhibition With DS and DDC

**Figure 2.**
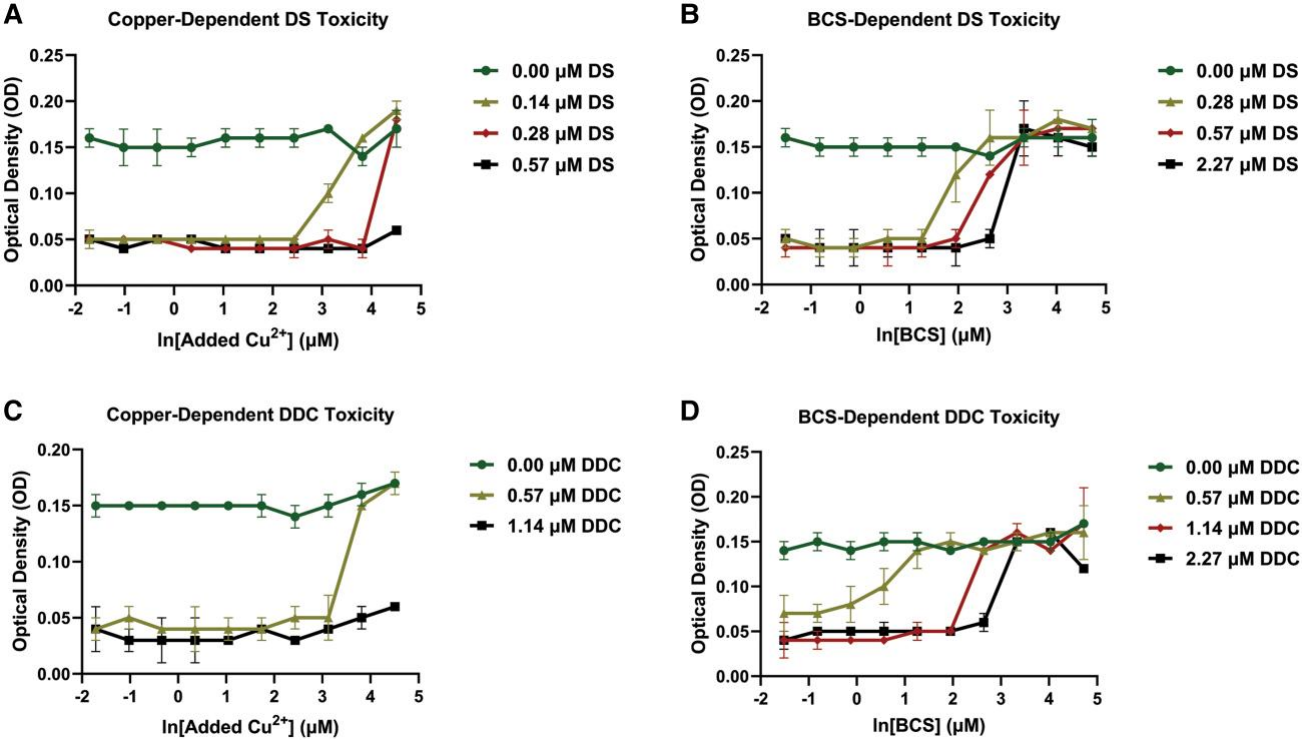
Copper-dependent *Mycoplasma genitalium* (Mgen) growth inhibition by disulfiram (DS) and diethyldithiocarbamate (DDC). *A*, Copper (Cu^2+^)–enhanced toxicity of DS as determined by Mgen growth inhibition in culture with different concentrations of Cu^2+^ and DS. Increased growth results in a decrease in pH with resultant color change measured by increasing optical density (450 nm). *B*, Dose-dependent reversal of DS inhibition of Mgen growth by bathocuproine sulfonate (BCS) at the indicated concentrations of each with no added copper (0.2 µM in the medium). *C*, Copper-dependent toxicity of DDC measured by Mgen growth inhibition in culture with the indicated concentrations of each. *D*, Dose-dependent reversal of DDC inhibition of Mgen growth by BCS with no added copper (0.2 µM in the SP4 medium). Values are mean ± SD for duplicate plates. Representative results of 3 experiments.

Mgen strain G37 was used to explore the mechanism of growth inhibition with DS seen with MIC testing (Table 1). Studies were conducted in SP4, which contains about 0.2µM Cu^2+^ [10]. The effect of Cu^2+^ concentration on DS antimicrobial activity is shown in Figure 2*A*. While the addition of DS at several concentrations (0.14, 0.28, 0.57 µM) resulted in growth inhibition, the growth of Mgen was not affected by the addition of up to 90µM Cu^2+^ in the absence of DS (0 µM). However, with higher concentrations of added Cu^2+^, the inhibitory effect of DS was lost in a dose-dependent manner. Relative to 0.57µM DS, less added Cu^2+^ was required to prevent Mgen growth inhibition at 0.14 and 0.28µM DS, respectively. To rule out the possibility that growth-inhibited wells contained viable bacteria, wells were recultured by transferring 10 µL to a fresh well containing 100 µL of fresh SP4 medium. There was no growth in wells without growth in the initial assay.

To explore the copper-dependent toxicity of DS, we used BCS, a highly specific copper chelator that is non–cell permeant, which directly competes with DS for Cu^2+^ binding (Figure 2*B*). While the addition of DS at several concentrations (0.28, 0.56, 2.27 µM) resulted in Mgen growth inhibition, this effect was lost with increasing concentrations of BCS. Less BCS was required to see normal growth at lower concentrations of DS. Mgen growth was not inhibited by the addition of up to 112µM BCS in the absence of DS (0 µM).

Since the copper-dependent effects of DS are due to copper complexes with the reduced monomer DDC [7], we examined the toxicity of different concentrations of added copper with DDC (Figure 2*C*). As seen with the parent compound DS (Figure 2*A*), the inhibitory effect of DDC was lost in a dose-dependent manner at higher concentrations of added Cu^2+^. Similar to DS (Figure 2*B*), the addition of BCS to wells containing DDC reduced the effectiveness of DDC in inhibiting growth (Figure 2*D*).

Consistent with the fact that each molecule of DS yields 2 molecules of the active metabolite DDC, the dose-response curves show that DDC was less potent at any given concentration while still exhibiting the same dose-response relationship for copper-dependent toxicity (Figure 2).

### ^64^Cu Uptake With DS and DDC

**Figure 3.**
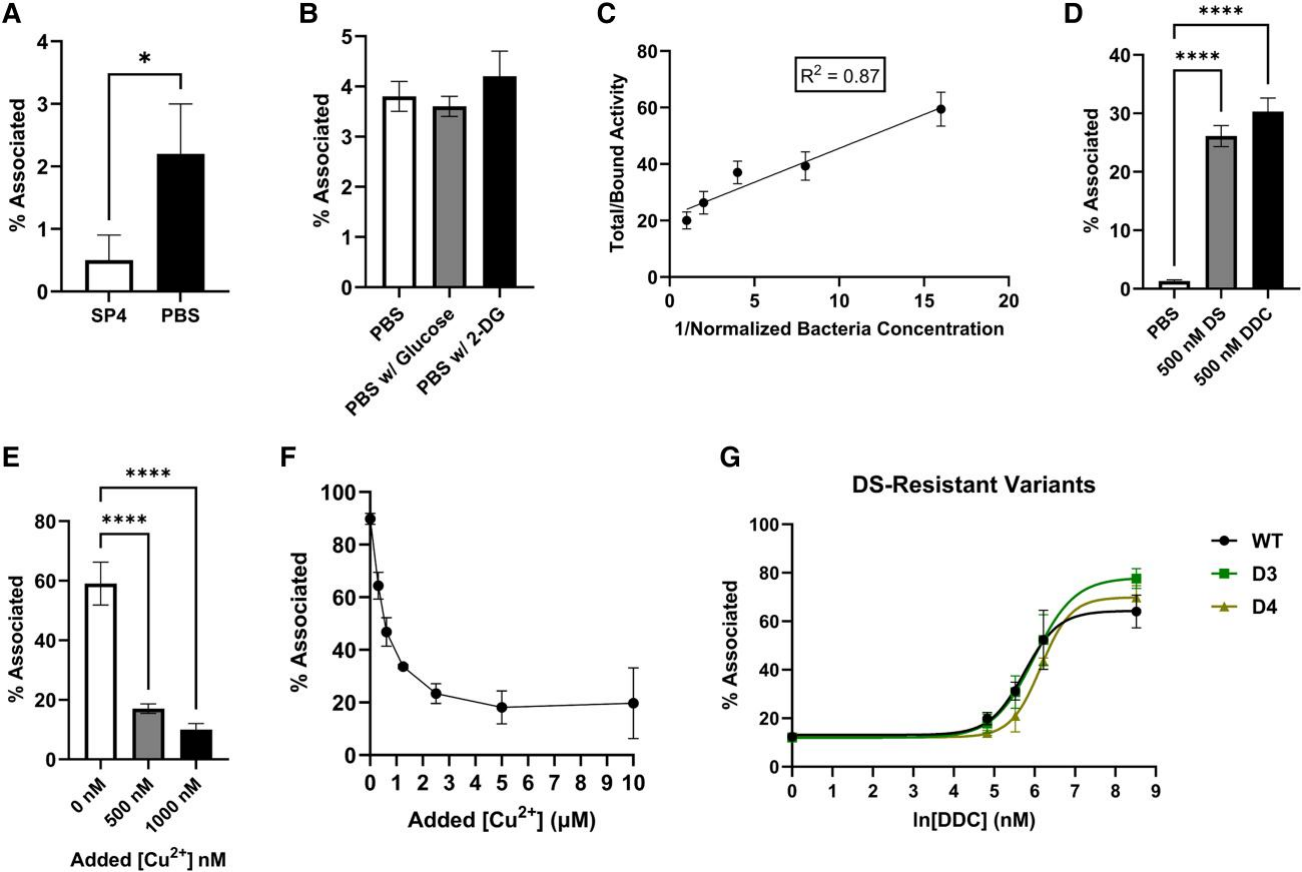
Copper (^64^Cu) uptake in Mgen reference strain G37. *A*, Uptake ^64^Cu (percentage associated tracer) in SP4 broth vs PBS. *B*, Uptake of ^64^Cu (percentage) in PBS with 25mM glucose and 50mM 2-DG vs PBS alone. *C*, Relationship between G37 concentration and ^64^Cu uptake in PBS. Starting with an initial bacterial suspension, four 2-fold serial dilutions were performed, and the reciprocals of the normalized concentrations are displayed on the y-axis. *D*, Uptake of ^64^Cu (percentage) with 500nM DS and 500nM DDC relative to PBS alone. *E*, Uptake of ^64^Cu (percentage) in PBS with 250nM DS with 0, 500, and 1000 nM of added CuSO_4_. *F*, Uptake of ^64^Cu (percentage) in PBS with 250nM DDC with 0 to 10 000nM CuSO_4_. *G*, Dose-dependent uptake of copper (Cu^2+^) by Mgen reference strain G37 (wild type [WT]) and 2 DS-resistant mutant Mgen strains (D3 and D4) in the presence of excess Cu^2+^ (500 nM) and the indicated concentrations of DDC in PBS. Values are reported as mean ± SD (n = 3 for each). *P < .05. ****P < .0001. DDC, diethyldithiocarbamate; DS, disulfiram; Mgen, *Mycoplasma genitalium*; PBS, phosphate-buffered saline.

Tracer concentrations of radioactive ^64^Cu were used to explore the effects of DS and DDC on Cu^2+^ uptake. To assess the likely possibility that excess Cu^2+^ in the SP4 broth at 0.2µM Cu^2+^ [10] would competitively reduce the ^64^Cu uptake produced in tracer concentrations of 10^−9^ M, Mgen suspensions were incubated with ^64^Cu in SP4 or PBS for 1 hour at room temperature (Figure 3*A*). We found that uptake of ^64^Cu was significantly reduced (*P* = .03) to 0.5% ± 0.4% associated in SP4 broth as compared with 2.2% ± 0.8% associated in PBS. Considering the possibility that Cu^2+^ uptake was energy requiring, Mgen was incubated for 1 hour with ^64^Cu in PBS with and without 25mM glucose, a metabolic substrate for glycolysis, and 50mM 2-deoxy-D-glucose, a glucose analog that competitively inhibits glycolysis (Figure 3*B*). As compared with PBS alone (3.8% ± 0.3% associated), there was no change in ^64^Cu uptake with the addition of 25mM glucose (3.6% ± 0.2% associated; *P* = .78) or 50mM 2-deoxy-D-glucose (4.2% ± 0.5% associated; *P* = .41). It was further shown that the uptake of ^64^Cu was directly proportional to the number of bacteria, with a strong positive correlation (*R*^2^ = 0.87) between ^64^Cu uptake and bacterial concentration (Figure 3*C*). Together these results suggest that ^64^Cu uptake was reduced in complex bacterial medium with excess Cu^2+^, not energy limited at the 1-hour time point, and directly proportional to the number of bacteria.

To evaluate the effect of DS and its metabolite DDC on ^64^Cu uptake, Mgen suspensions were incubated with 500nM DS or DDC in the absence of additional copper (Figure 3*D*). When compared with PBS alone (1.3% ± 0.2% associated), there was a significant increase in ^64^Cu uptake with the addition of 500nM DS (26% ± 1.8% associated; *P* < .0001) and with the addition of 500nM DDC (30% ± 2.3% associated; *P* < .0001). The difference in uptake between 500nM DS and DDC (*P* = .052) was not significant but trended higher with 500nM DDC.

Furthermore, the addition of excess free Cu^2+^ in the form of CuSO_4_ significantly reduced ^64^Cu uptake in the presence of DS (Figure 3*E*) and DDC (Figure 3*F*). The addition of 500nM CuSO_4_ (17% ± 1.6% associated) and 1000nM CuSO_4_ (10% ± 2.0% associated) significantly reduced (*P* < .0001) the uptake of ^64^Cu as compared with DS alone (59% ± 7.2% associated). The difference in uptake between 500 and 1000nM CuSO_4_ was not significant (*P* = .21). ^64^Cu uptake was also reduced by the addition of excess Cu^2+^ following incubation with 250nM DDC, from 90% ± 2.1% associated with DDC alone to 19.7% ± 13.4% associated with 10mM added Cu^2+^, in a saturable mechanism. These results confirm the effect of DS and DDC in greatly enhancing copper uptake by the bacteria, as supported by the competitive inhibition of ^64^Cu uptake by the addition of excess Cu^2+^.

As shown in Figure 2, DS and DDC toxicity against Mgen is copper dependent. Thus, if DS were to be used as an antimicrobial against Mgen, it would be expected that it would not be effective in the presence of excess Cu^2+^. With ^64^Cu as a tracer, conditions of relative Cu excess ([Cu^2+^] >> [DDC]) and relative Cu dearth ([Cu^2+^] << [DDC]) were created by adjusting the concentration of DDC relative to a constant concentration of CuSO_4_ at 500 nM (Figure 3*G*). We applied these conditions to the DS-susceptible wild type (WT) parental G37 Mgen strain alongside 2 DS-resistant mutant sublines, D3 and D4, which tolerate DS at about 8 and 1 mg/L, respectively. As shown on the left of Figure 3*G*, uptake of ^64^Cu is low in the condition of relative Cu^2+^ excess ([Cu^2+^] = 500 nM, [DDC] = 1 nM), with 2.4% ± 0.3% associated in the WT G37 strain, 2.0% ± 0.8% associated in the D3 mutant, and 2.0% ± 1.3% associated in the D4 mutant. By contrast, in the condition of relative Cu^2+^ dearth ([Cu^2+^] = 500 nM, [DDC] = 5000 nM), uptake of ^64^Cu is high, with 64% ± 6.8% associated in the WT G37 strain, 78% ± 4.1% associated in the D3 mutant, and 70% ± 4.9% associated in the D4 mutant (Figure 3*G*, right). Importantly, ^64^Cu uptake is not affected in the D3 and D4 mutant strains as compared with the WT, despite evidence of DS resistance in these mutant strains.

## DISCUSSION

Although DS was established in the 1950s as a treatment for alcoholism, its use in clinical practice has fallen in the last 2 decades. In more recent years, DS has undergone evaluation as a possible therapeutic option in the treatment of other addictions, such as cocaine use disorder; as an antimicrobial treatment for bacterial, fungal, viral, and parasitic diseases; and in the treatment of malignancies and inflammatory diseases such as Graves disease [7]. It exerts its effects through several important mechanisms, including direct inhibition of enzymes, chelation of important metal enzyme cofactors, and generation of reactive oxygen species [6, 7, 14–16]. DS itself, when reduced by free sulfhydryl groups of proteins via the thiol-disulfide exchange reaction, can irreversibly modify the cysteine residue on target enzymes, resulting in their inhibition [7].

**Figure 4.**
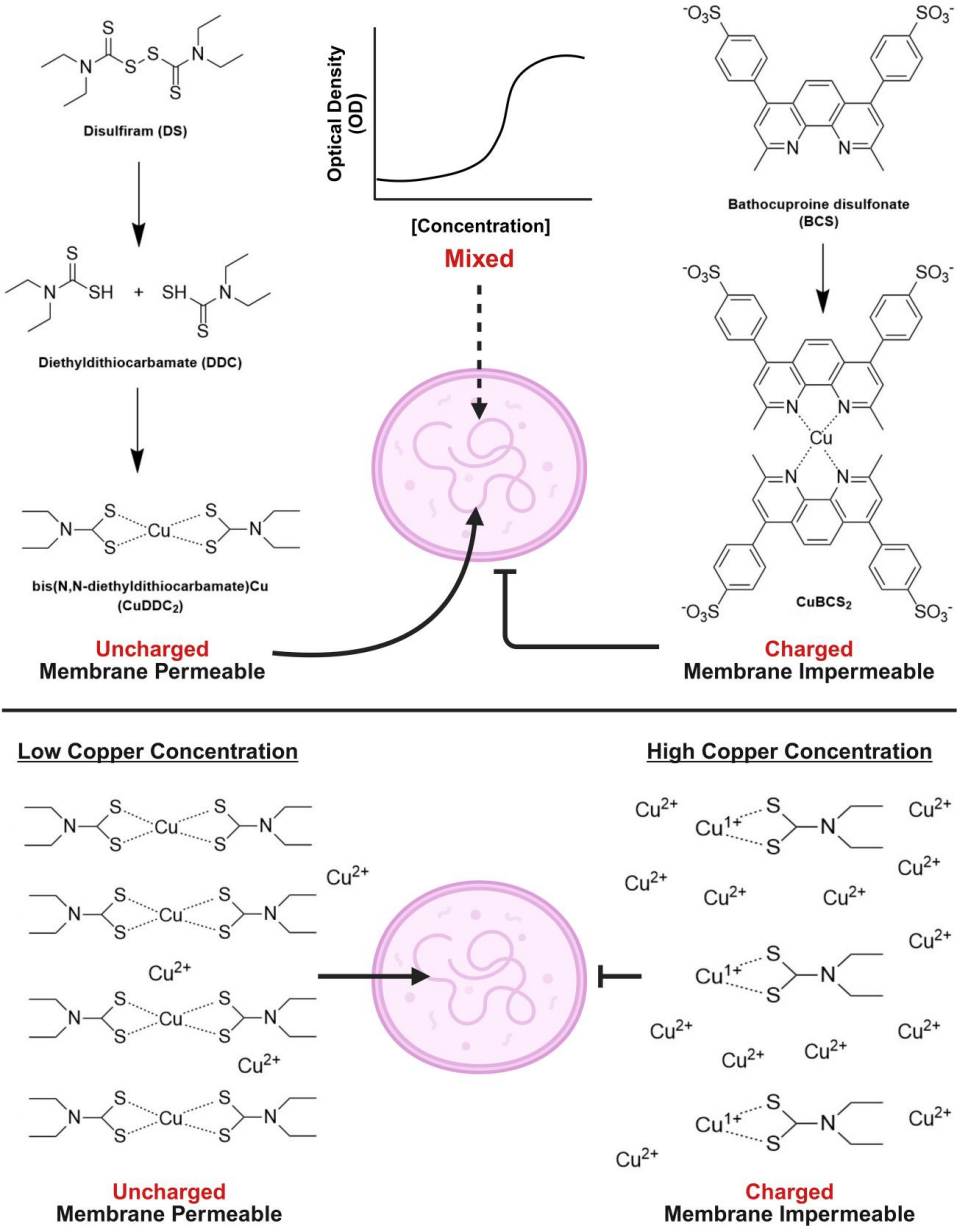
Modeling the mechanism of DS/DDC-mediated copper uptake. Upper panel: DDC formed by the reduction of the prodrug DS forms membrane-permeant complexes with copper under appropriate molar ratios. The water-soluble copper-specific chelator bathocuproine binds copper but is membrane impermeant. Lower panel: Complexes with copper formed by DDC under low copper concentrations are membrane permeable while those formed at high copper concentrations are not. DDC, diethyldithiocarbamate; DS, disulfiram.

In this study, we found that Mgen has the highest reported sensitivity to DS of any bacteria studied thus far [17] and that this sensitivity to DS is copper dependent. One peculiarity about the copper-dependent toxicity of DS and DDC is the inverse correlation with the copper concentration. This effect has been reported in a study of DS-induced toxicity and radiosensitization of cancer cells, where the authors noted that “the concentration of copper relative to disulfiram is a major determinant of disulfiram's potency” [18]. In studies with *Staphylococcus aureus* and *S epidermidis*, Kaul et al observed low bacterial killing when DDC concentrations exceeded Cu^2+^ concentrations, suggesting that the molar ratio of DDC:Cu^2+^ is important in killing [19]. In experiments with *Mycobacterium tuberculosis*, Dalecki et al observed optimal growth inhibition with 0.6µM DDC and 0.3µM Cu^2+^, a 2:1 ratio [20]. In our experiments, we saw growth inhibition with 570nM DDC with no added copper, about 200nM copper in the SP4 medium, for a ratio of 2.85:1, close to that seen by Dalecki et al. These results in eukaryotic cells and bacteria imply that the mechanism of copper dependency relates to the chemistry of DDC's interaction with copper. We speculate that at higher concentrations of copper, the DDC-Cu^2+^ complexes that form are charged and hence cell impermeant, while at low copper concentrations, the complexes are neutral and readily membrane permeant. A proposed model for this phenomenon is shown in Figure 4. The formation of neutral complexes of 2 DDC molecules with Cu^2+^ is a known property of DDC [20]. If DDC-copper complexes are necessary for DDC and copper to enter the cell, the subsequent toxicity in bacteria and eukaryotic cells could depend on either DDC or copper or both, and the mechanism of toxicity might be quite different in cancer cells and microbes.

Our studies with ^64^Cu demonstrate direct DS/DDC-mediated uptake of Cu with Mgen that is far above the uptake observed in the absence of either drug. This is consistent with the “Trojan horse” model proposed by Dalecki et al in which Cu(DDC)_2_ complexes shuttle Cu^2+^ into the cytoplasm, shielding it from the bacterial resistance mechanisms [20]. However, ^64^Cu uptake did not explain the DS resistance seen in mutant D3 and D4 strains as compared with WT. We infer that the DS resistance seen in the sublines is not due to a difference in DS-mediated Cu entry into the cell or by expulsion from the cell (eg, by an efflux pump) but instead is likely due to a mutation in a gene or genes through which copper-dependent toxicity occurs. It must also be considered that once inside the cell DDC itself may be toxic in some way to Mgen, perhaps by inhibition of glycolysis [21], which, as in the closely related *M pneumoniae*, is the major pathway for generating ATP in these bacteria [22].

The high degree of sensitivity of Mgen for DS/DDC with an organism's ecologic niche in the urogenital tract brings up the possibility that DS might be useful clinically, at least as adjunctive antimicrobial therapy for Mgen. Older studies have demonstrated that the major portion of radiolabeled DS, though excreted in the urine, is excreted primarily as inactive metabolites [7, 9, 23]. However, Linderholm and Berg showed in 1951 in human volunteers that if the urine is alkalinized by coadministration of sodium bicarbonate, 1.6% of the administered DS is excreted in the urine as DDC. Faiman et al analyzed the kinetics of DS and metabolites in the plasma of human volunteers following a 250-mg oral dose [24]. They found that DDC reached peak plasma concentrations in 8 hours with the majority of the dose eliminated in 24 hours. Thus, although only a small proportion of a dose would be excreted as DDC, it could be concentrated to the point of pharmacologic efficacy against Mgen. In addition, rat studies demonstrated that the kidney was the organ with the highest tissue concentration of radiolabeled DS metabolites [25]. The reference range for 24-hour urine copper excretion is 300 to 800 nmol, which gives a copper concentration of 200 to 540 nM in a daily urine volume of 1.5 L for an average adult, although of course this would vary widely. This concentration of copper is in the range of that in the SP4 medium used in this study. Only future clinical studies can determine whether the in vitro antimicrobial efficacy of DS/DDC against Mgen translates to efficacy in patients with drug-resistant Mgen infections.

Our study has limitations. First, we cannot confirm that drug-metal complexes are transported into the bacterial cells, only that there is concentration-dependent association of copper with the bacteria in the presence of the drug. Our observation of the inverse correlation of copper concentrations with drug-induced growth inhibition has been noted in studies in eukaryotic cells and bacteria [18–20]. We have attempted to explain this observation with a hypothetical model shown in Figure 4. An alternative possibility might be that DDC binds another trace metal as the copper concentration drops, and it is this metal that actually causes the toxicity. However, the reversal of DS and DDC toxicity by the highly copper-specific chelator BCS strongly supports the idea that the toxicity of these compounds is copper dependent. Furthermore, at least for zinc, Dalecki et al demonstrated spectrophotometrically that DDC-Cu complexes form even when commercial DDC-Zn was used as the DDC source, indicating that copper outcompetes zinc for binding to DDC [20]. Finally, a practical limitation for the applicability of our findings in the treatment of multidrug-resistant Mgen infections might be an inability to achieve appropriate concentrations of DDC and Cu^2+^ in the patient's urine.

In summary, we demonstrate that DS and its metabolite DDC exhibit potent copper-dependent antimicrobial activity against Mgen. The possibility that the excretion of small amounts of active DDC with free copper in the patient's urine might suffice to provide antimicrobial activity against Mgen will need to be explored in future in vivo studies.
